# 7q36 deletion and 9p22 duplication: effects of a double imbalance

**DOI:** 10.1186/1755-8166-6-2

**Published:** 2013-01-15

**Authors:** Karla de Oliveira Pelegrino, Sofia Sugayama, Ana Lúcia Catelani, Karina Lezirovitz, Fernando Kok, Maria de Lourdes Chauffaille

**Affiliations:** 1 Research and Development Institute, Fleury Group, Av. Gal Valdomiro de Lima, 508, São Paulo, SP, Zip Code: 04344-070, Brazil; 2 Pediatrics Department, Hospital das Clínicas, Faculdade de Medicina, University of Paulo, São Paulo, SP, Brazil; 3 Departamento de Otorrinolaringologia (LIM32), Hospital das Clínicas, Faculdade de Medicina, University of São Paulo, São Paulo, SP, Brazil

**Keywords:** Developmental delay, 7q deletion, 9p duplication, aCGH array

## Abstract

The etiology of mental retardation/developmental delay (MRDD) remains a challenge to geneticists and clinicians and can be correlated to environmental and genetic factors. Chromosomal aberrations are common causes of moderate to severe mental retardation and may represent 10% of these occurrences. Here we report the case of a boy with development delay, hypoplasia of corpus callosum, microcephaly, muscular hypotonia, and facial dysmorphisms. A deletion of 7q36.1 → 36.3 and duplication of 9p22.3 → 23 was detected as a result of an unbalanced translocation of paternal origin. Breakpoint delimitation was achieved with array comparative genomic hybridization assay. Additional multiplex ligation dependent probe amplification (MLPA) analyzes confirmed one copy loss of 7q36.3 region and one copy gain of 9p24.3 region. Patient resultant phenotype is consistent with the already described findings for both 7q deletion and 9p duplication syndromes.

## Background

Mental retardation/developmental delay (MRDD) is a relevant public health concern, since it may represent one of the major causes of child disability, with prevalence estimated in 1-3% [1]. In Brazil, the Governmental Census conducted in 2010 estimated that MRDD can be found in 1.4% of population [2], but this number may be underestimated. Actually, there are deficiencies not only in evaluating MRDD prevalence around the world but also in methodology for screening and impairment degree stratification [3,4]. MRDD causes are heterogeneous and may have both environmental and genetic bases. It is estimated that 65-80% of patient’s MRDD etiology remains unknown [5].

9p duplication syndrome is considered one of the most frequent autosomal anomalies in life born together with trisomies 13, 18 and 21 [6]. More than 150 cases of 9p duplication are reported in literature [7] and common features include mental retardation, failure to thrive, hypotonia, microcephaly, low set malformed ears, upward-slanted eyes, small palpebral fissures, congenital heart disease, skeletal and genitourinary anomalies, enophthalmos or microphthalmos, broad base nose and prominent nasal tip, downward slanting mouth, among others characteristics [7,8].

Similarly, deletion of 7q is a described syndrome [9] and clinical findings comprise low birth weight, mental retardation, development delay, facial dysmorphisms and genitourinary malformations [10-19]. Additionally, more severe phenotypes, as sacral agenesis and holoprosencephaly (HPE), may occur [9,14-17,20]. Here we report the case of a boy exhibiting 7q deletion and 9p duplication due to an unbalanced translocation t(7;9) from paternal origin.

## Case presentation

The patient (male) is the first child of a 31-year-old mother and her unrelated 28-year-old husband. Mother was Caucasian and father Japanese descent. Ultrasonography on 33 weeks of gestation revealed intrauterine growth retardation (IUGR) and diminished vascular sport. The mother reported only vitamins ingestion during pregnancy. The proband was born by cesarean section (37 weeks), Apgar were reportedly 5-8, weight of 1950 g, length of 41 cm and occipitofrontal head circumference 29.0 cm.

The newborn presented peculiar facies, severe microcephaly, and generalized hypotonia. He also developed jaundice without ABO incompatibility and phototerapy was required. Echocardiography with 2 days of life disclosed normal heart function. At the age of 5 months the child weight (5345 g), length (58 cm) and occipitofrontal head circumference (36.5 cm) were below the second percentile. Dysmorphic features included anterior fontanel with dimension like one finger, bilateral epicanthal folds, upslanting palpebral fissures, bulbous nasal tip, enlarged columela, large symmetric and posteriorly rotated ears. Limbs, hands and feet were symmetric. Hallux vagus and overlapping of the left third toe on the second toe were observed. Finger and toe nails were hyperconvex. Thorax and lombo-sacral radiographies revealed no abnormalities.

**Figure 1:**
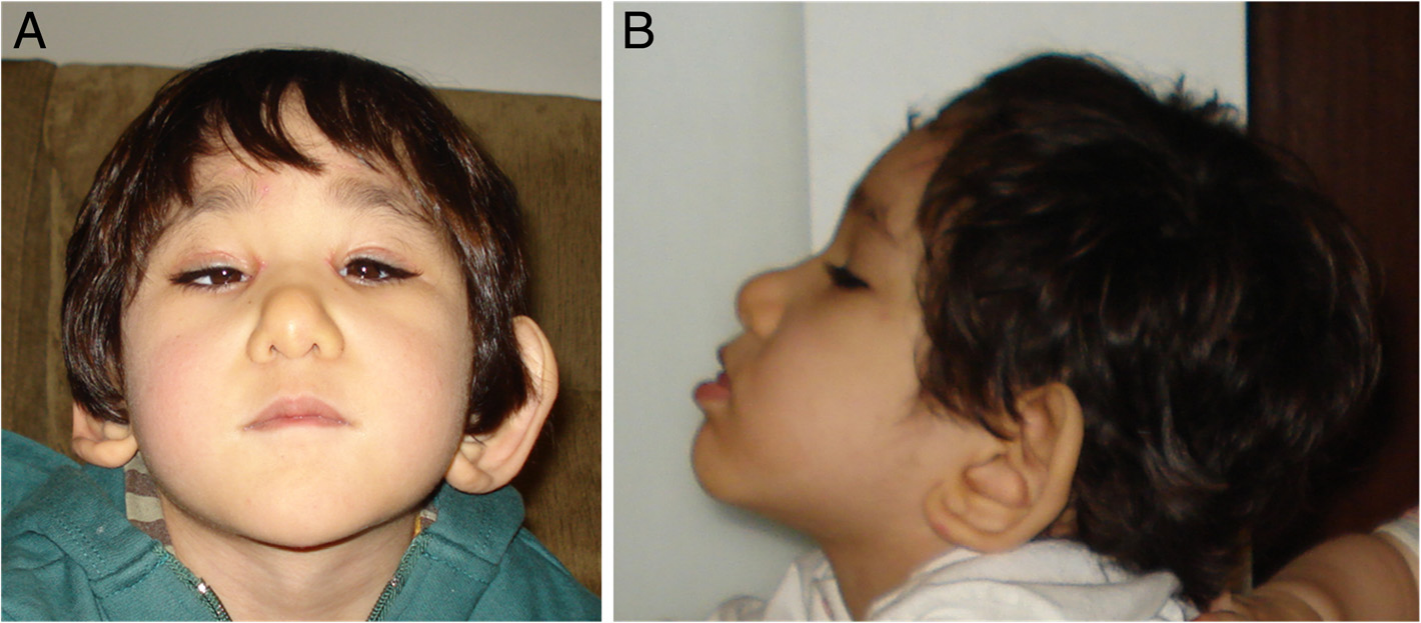
**Patient phenotype at 5 years-old.** Frontal (**A**) >and side (**B**) view

At 2 years old, patient presented seizure without fever (10 minutes duration) while sleeping. EEG disclosed focal epileptiform activity expanding to bilateral fronto central and medial region, with left side predominance. MRI scan of the brain showed hypoplasia of corpus callosum and white matter reduction. At age of 3 years old and 5 months child exhibited failure to thrive (weight = 9.5 kg, p < 3; length = 86.0 cm, 9p < 5), psychomotor and language delay, severe microcephaly (OFC = 43.5 cm, p < 3) and craniofacial dysmorphisms became more evident. Patient also presented micropenis (3.5 cm (p < 10). Patient phenotype at 5 years old can be assessed in Figure 1.

**Figure 2:**
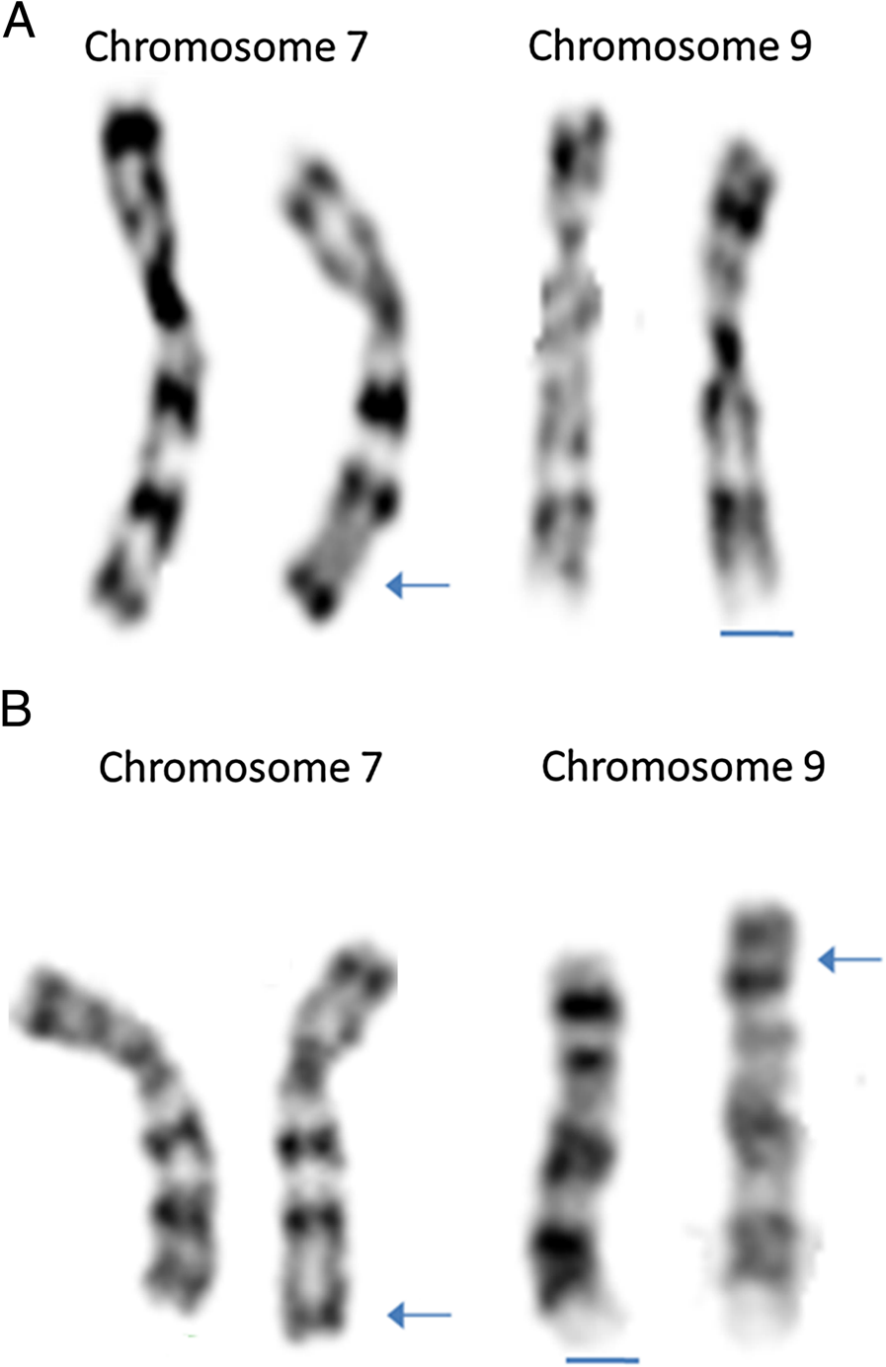
**Partial G banded karyotypes of the proband** (**A**) **and father** (**B**)**.** Arrows indicate regions of translocation and bars 9 ph variation

Peripheral blood karyotype (Phytohemagglutinin -PHA-stimulated lymphocyte with G banding-500 bands) from propositus and progenitors were studied. Abnormalities were described according to ISCN (2009) [21]. Father analysis showed a 46, XY, der(7)t(7;9)(q36;p22), 9 ph karyotype, while none structural or numeric alterations were observed for mother. Patient karyotype was 46, XY, der(7)t(7;9)(q36;p22), 9 ph (Figure 2).

Additional aCGH and MLPA investigations were conducted. Patient and progenitors DNA samples were extracted from blood with Qiamp Dna Blood Midi (Qiagen, Germany). aCGH procedures were performed as recommended by manufacturer (Perkin Elmer, Norwalk). Constitutional Chip 4.0 (Perkin Elmer, Norwalk) was employed and was comprised of 5000 BAC clones spotted in duplicate, with resolution lower than 650 kb and segments arranged from 100 to 300 kb of human DNA, distributed through whole genome.

Slides were scanned with InnoScann 710 (Innopsys, Carbonne, France) and MAPIX 4.5 software was used for GPR files generation. Data analysis was conducted with SpectralWare® v2.3.3 aCGH Analysis System software [22] from Perkin Elmer. The computation parameters used were pin linear for normalization; threshold between 0.7/1.3; Lowess alpha of 0.1 normalization and confidence level of 95%. Median data were used for interpretation of results. For chromosome region size determination and CNV search, Database of Genomics Variants was consulted [23]. Analyses of proband DNA revealed a 7q deletion (7q36.1 -- > qter) and 9p duplication (9p22.3-- > 23) (Table 1). In 7q deleted segment, 80 genes are present, although the BAC clones of aCGH array comprised less than 20% of them. In a similar way, duplicated region of 9p encloses 42 genes, however only two genes were detected through hybridization with BAC clones of the chosen platform. None alteration was observed in maternal DNA analysis. Losses of two clones (14q32.33 and 16p13.13) were detected in paternal DNA analysis, both unrelated to child’s phenotype.

**Table 1:** Clones that presented copy number variation detected through aCGH screening of patient genome

**Clones**	**Status of alteration**	**Cytogenetic location**	**Genomic coordinates** (**GRCh36 hg18**)	**Genes present in the region**
RP11-43 L19	Loss	7q36.1	151,167,622: 151,325,223	*PRKAG2*, *GALNTL5*, *LOC100505483*
RP11-958 M14	Loss	7q36.2	152,448,407: 152,551,324	-
RP11-79 K9	Loss	7q36.2	153,634,608: 153,780,253	*DPP6*
RP11-80 J22	Loss	7q36.2	154,256,381: 154,418,755	*DPP6*, *LOC100132707*, *PAXIP1*
RP11-69O3	Loss	7q36.3	155,193,647: 155,348,385	*RBM33*, *SHH*
RP11-264P5	Loss	7q36.3	155,707,993: 155,735,684	-
RP11-260H17	Loss	7q36.3	155,945,431: 156,046,133	*LINC00244*
RP11-58 F7	Loss	7q36.3	157,265,524: 157,458,433	*PTPRN2*, *LOC100506585*
RP11-230 K23	Loss	7q36.3	157,779,951: 157,964,008	*PTPRN2*
GS-3-K23	Loss	7q36.3	158,551,928: 158,650,980	*VIPR2*
RP11-343D17	Gain	9p23	9,188,596: 9,374,041	*PTPRD*
RP11-32D4	Gain	9p23	11,469,225:11,641,680	-
RP11-79B9	Gain	9p23	14,010,094:14,171,677	*NFIB*
RP11-109 M15	Gain	9p22.3	16,141,129:16,325,493	-

**Figure 3:**
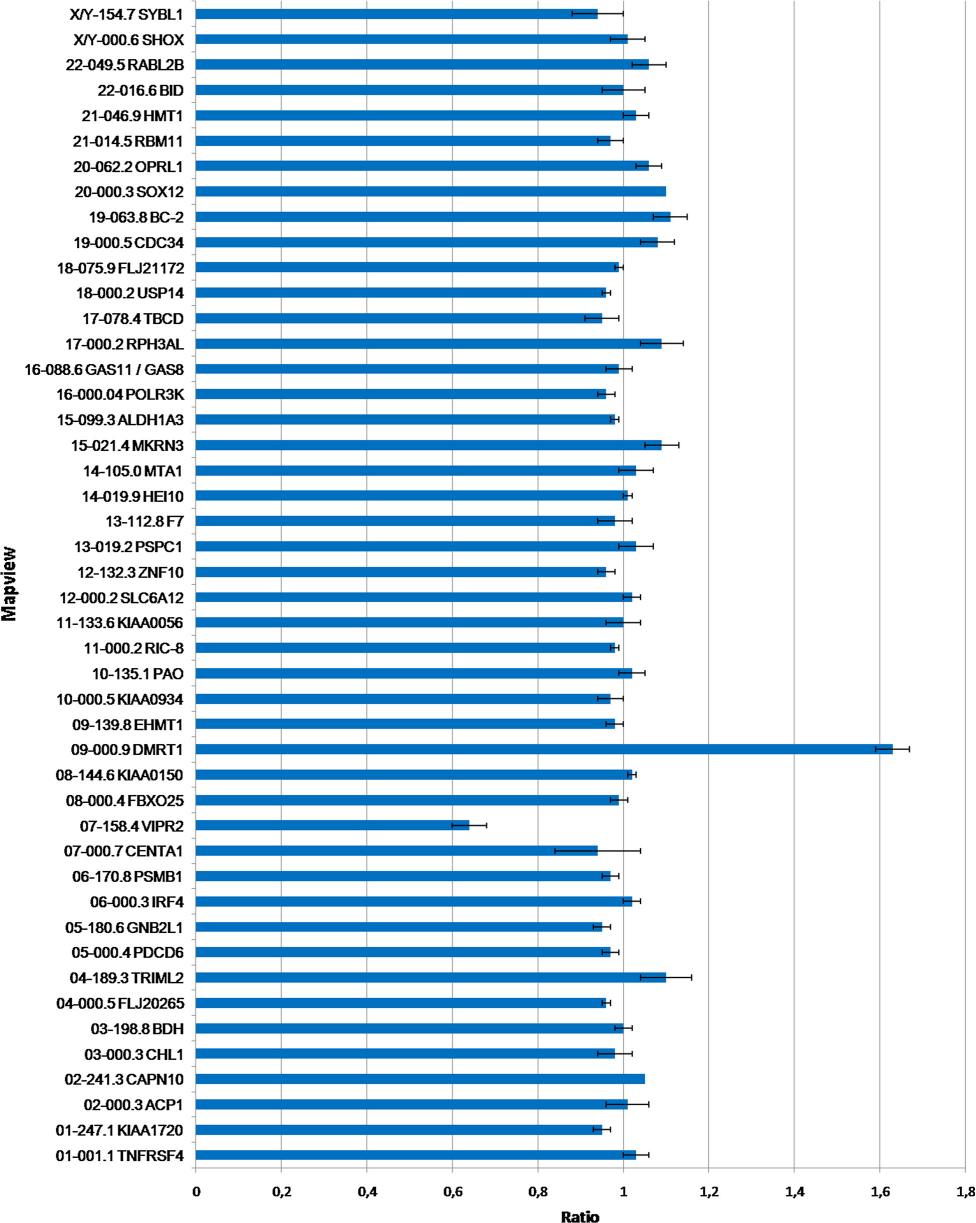
**MLPA ratio chart of proband.** Analysis revealed gain for gene DMRT1 in 9p24.3 region (1,63+/-0,04) and loss for gene VIPR2 (0,64+/-0,04) located in 7q36.3

Multiplex ligation-dependent probe amplification (MLPA) assay was performed with SALSA MLPA P036-E1 Human Telomere-3 probemix kit (MRC-Holland; Amsterdam, The Netherlands). Reaction was conducted as recommended by manufacturer. Fragments were separated on an ABI3100 genetic analyzer (Applied Biosystems). Genotyper Software (version 2.0) and Coffalyser V9.4 Software [24] were employed for data analyses. None alteration was observed for both parents. One copy loss of VIPR2 gene (7q36.3) and one copy gain of DMRT1 gene (9p24.3) were detected in the patient DNA analysis (Figure 3).

## Discussion

Deletion of terminal portion of chromosome 7 long arm is an established syndrome. More than 50 cases are reported and in most of them deletion of 7q32 → qter is involved [20,25]. Microcephaly, short stature, mental and growth retardation, alobar and (semi)lobar HPE, facial dysmorphisms and genital hypoplasia in males are generally observed [9]. Clinical findings also include malformed ears, hypoplasia of corpus callosum, abnormal fingers, overriding toes, caudal deficiency sequence and chest abnormalities, as pectum excavatum [12,25]. Ocular defects are also described [12,19,26-28]. Table 2 summarizes data of 8 patients with one copy deletion of 7q as well as those of present case.

**Table 2:** Clinical manifestations of 7q deletion cases

**Case**	**Present case**	**Frühmesser *et al***. **2012**[10]	**Ponnala and Dalal**, **2011**[11]	**Vorsanova *et al***. **2008**[12]	**Ahn *et al***. **2003**[13]	**Nowaczyk *et al***. **2000**[14]	**Chen *et al***. **1999**[15]	**Savage *et al***. **1997**[16]	**Hara *et al***. **1986**[19]
*Karyotype*	46,XY, der (7), t(7;9) (q36;p22), 9 ph	46,XX, der(7), t(7;17) (q36;p13)	46, XY, der (7), t(7;14), (q33-q32.2)	45,XX, der(7), t(7;21) (q34;q22.13),-21	46,XX, der(7), t(2;7) (q37.3;q34)	46,XY, der(7), t(2;7) (p23.2;q36.1)	46,XX, der(7), t(3;7) (p23;q36)	46,XX, der (7), t(7; 19) (q36.1;q13.43)	46, XX, der(7), t(5;7) (q31:q22)
*Origin*	Paternal	Maternal	Paternal	*de novo*	Paternal	Paternal	Paternal	Paternal	Uncertain
*Age at diagnosis*	5 months §	21 years §	4 years	2 years §	fetus	fetus §	fetus §	fetus §	1 year
*Developmental delay*	+	+	+	+					+
*Mental retardation*		+		+					+
*Hypotelorism*				+					
*Microftalmia*						+			
*Microcephaly*	+		+	+					+
*Up slanting palpebral fissures*	+			+	+				
*Malformed ears*	+		+	+	+				
*Broad*/*bulbous nasal tip*	+		+	+					+
*Arrhinencephaly*							+		
*Cleft lip*					+				
*Cleft palate*					+				
*Nail hypoplasia*	+								
*Hallux vagus*	+								
*Overlapping toes*/*fingers*	+		+						
*Fingers malformations*	+	+		+					
*Multiple skeletal abnormalities*		+		+					
*Sacral agenesis*							+	+	
*Urogenitally abnormalities*	+					+			
*Epileptic seizures*	+								
*HPE*						+	+		
*Hypoplasia of the corpus callosum*	+	+							
*Ciclopia*							+		
*Muscular Hypotonia*	+	+	+	+					
*Cardiac defetcts*				+					
*Maternal age at conception*	31	30	not informed	24	43	not informed	26	29	35

§Intrauterine growth retardation was reported.

Although HPE was not pointed as a feature of the case here shown, MRI scan of the brain of the patient disclosed hypoplasia of corpus callosum, which may be considered a minimal finding of HPE [20]. In fact, 7q36.3 region encloses the gene *SHH*- *Sonic Hedgehog*, which haploinsufficiency was previously associated with HPE [29,30], microcephaly and cerebral midline defects [31]. Some characteristics as mental retardation, microcephaly, hypotonia and failure to thrive are common features of 9p duplication syndrome, also exhibited by this patient.

Duplication of 9p was recognized as a syndrome in 1970 [8], since then, more than one hundred cases were described and additional findings comprise mental retardation, failure to thrive, hypotonia, microcephaly, hypertelorism, deep set eyes in non-horizontal position, cup-shaped ears, kyphoscholiosis, cryptorchidism in males and syndactily of toes [32]. Moreover, 9p duplication was already associated to HPE [33] and epilepsy [34], also observed in this case.

In fact, double imbalances, as the present one, may confer phenotypic variability to described syndromes, turning it difficult to predict the characteristics that evolved as a result of the global gene imbalance, caused by the concomitant duplication and deletion, and the contribution of each isolated alteration to resulting phenotype.

Actually, chromosomes 7 and 9 are grouped among those frequently involved in karyotype alterations leading to pregnancies losses, mental retardation and multiple congenital abnormalities [35]. Chromosome 7 is also observed as one of the three preferentially involved in reciprocal [36] and complex translocations [37], while 9p23-24 is suggested as hotspot for chromosome breaking [38].

Interestingly, reciprocal translocation t(7;9) leading to 7q33 → qter duplication and pter → 9p23 deletion was also described and phenotype involved prominent metopic suture, epicanthal folds, strabismus, low-set ears, microretrognathia, large anterior fontanel, bilateral simian creases, muscular hypotonia, severe psychomotor retardation [39] and hermaphroditism [40]. Additional cases may help to evaluate if t(7;9) is a recurrent translocation correlated to miscarriages, MRDD and congenital birth defects.

Aneuploidy together with segmental aneusomy constitute the mainly cause of pregnancy losses in humans. Additionally, among 5% of all pregnancies present aneusomies [41] and the resulting children can show malformations and MRDD. It is estimated that 1 out of 150 liveborn babies present chromosomal abnormalities [42], but this number might rise with the employment of arrays and next generation sequencing. In fact, the advent of these high throughput techniques has permitted whole genome scanning with finer resolution, leading to detection of subtelomeric rearrangements and microalterations previously undetectable through conventional approaches, as G-band karyotyping and FISH [43]. Furthermore, DNA arrays have been useful for breakpoint site determination and copy number evaluation improving correlation among chromosomal region alteration and resulting phenotype.

## Conclusions

We present the case of a patient exhibiting deletion of 7q36.1 → 36.3 and duplication of 9p22.3 → 23 as a result of an unbalanced translocation from paternal origin. Breakpoint delimitation was achieved with array comparative genomic hybridization analyzes and MLPA assays confirmed the alterations found. Patient presented development delay and facial dysmorphisms, among other abnormalities, consistent with both 7q deletion and 9p duplication syndromes. Reciprocal translocation leading to 9p deletion and 7q duplication had already been reported in two unrelated cases, indicating that t(7;9)(q36;p23) may occur more frequently than expected. Further cases may confirm these results. Finally, the increasing use of high throughput platforms for genomic studies tends to refine chromosome breakpoint analyses, evaluate critical regions involved in genetic disorders and disclose micro rearrangements firstly inaccessible through conventional approaches.

## Consent

Written informed consent was obtained from patient’s progenitors for publication of this case report and any accompanying images. A copy of the written consent is available for review by the Editor-in-Chief of this journal.

## Competing interests

The authors declare that they have no competing interests.

## Authors’ contributions

PKO performed the aCGH experiments, MLPA assays, data analysis and interpretation, and participated in the preparation and revision of the manuscript. SS was responsible for the patient examination and clinical description, and participated in the preparation of the manuscript. CAL performed the karyotype analysis. LK performed the DNA extraction and participated in aCGH experiments. KF participated in writing of the discussion section and in the revision of the manuscript. CML coordinated the study, designed the project, was responsible for fund obtaining, and participated from data interpretation, manuscript writing and review. All authors read and approved the final manuscript.
